# Differences in risk factor profiles for peripheral artery disease compared to coronary, cerebral and carotid artery

**DOI:** 10.1038/s41598-025-88516-0

**Published:** 2025-01-31

**Authors:** Stefan Acosta, Yufeng Du, Yan Borné, Anders Gottsäter

**Affiliations:** 1 Department of Clinical Sciences, Lund University, Ruth Lundskogs g 10, 205 02 Malmö, Sweden; 2 Department of Cardiothoracic and Vascular Surgery, Vascular Center, Malmö, Sweden; 3 Institute of Epidemiology and Statistics, School of Public Health, Lanzhou University, Gansu, China; 4 Department of Acute and Internal Medicine 3, Skåne University Hospital, Malmö, Sweden

**Keywords:** Cardiology, Diseases, Risk factors

## Abstract

The aim of this study was to evaluate the greatest drivers for development of lower extremity peripheral artery disease (PAD) in relation to coronary, precerebral, or cerebral artery disease This prospective study (Malmö Diet and Cancer study) included 26,681 participants. The diagnosis of incident PAD, coronary artery disease (CoAD), atherothrombotic ischemic stroke (IS) free from atrial fibrillation or flutter, and carotid artery disease (CaAD) was validated. A modified Lunn-McNeil competing risk analysis was performed to compare the Hazard Ratio (HR) strength of PAD in relation to CoAD, IS, or CaAD. The estimated population attributable risk fractions (PAF) for each atherosclerotic manifestation were estimated by first fit an age and sex adjusted Cox proportional hazard regression, and then estimate the PAF using the Direct method. Male sex, age, and hypertension were risk factors for development of all atherosclerotic manifestations. Current smoking accounted for 45.6% (95% CI 41.1–47.2), 16.1%, 14.0%, and 23.3% of the risk for development of PAD, CoAD, IS, and CaAD, respectively. Hypertension was more associated with development of PAD than CoAD (*p* = 0.009). Smoking and diabetes mellitus were positively associated with all four manifestations, but these associations were significantly stronger for PAD than the other three manifestations. Smoking and diabetes mellitus had a larger impact on incident PAD than incident coronary, cerebral or precerebral artery manifestations. Since the lower extremity arteries are the easiest to access and examine, they may be considered as the first arterial bed to examine in patients at increased risk for atherosclerotic manifestations.

**Supplementary Information:**

The online version contains supplementary material available at 10.1038/s41598-025-88516-0.

## Introduction

Atherosclerotic cardiovascular disease (ASCVD) of the coronary and cerebral arteries are the two leading causes of death worldwide^1^. Conventional risk factor control such as smoking cessation, control of systolic blood pressure and total cholesterol reduces ASCVD burden, but the worldwide rise in obesity and diabetes mellitus threatens to counteract such preventive efforts^2^. This shift in risk factors may lead to that relative more women in the future will develop an ASCVD^3^.

Evaluation regarding differences in risk factor profiles between ischemic stroke and coronary events^4^, coronary events and abdominal aortic aneurysm^5^, lobar and non-lobar intra-cerebral haemorrhage^6^, Parkinson´s disease and coronary events, and Parkinson´s disease and ischemic stroke^7^ in the Malmö Diet and Cancer Study (MDCS) have been done, but no such evaluation has been performed regarding potential differences in risk factor profile between peripheral artery disease (PAD), coronary artery disease (CoAD), atherothrombotic ischemic stroke (IS), or carotid artery disease (CaAD). Even though these four different atherosclerotic manifestations share common risk factors, it is of great interest to have a better understanding of the greatest risk factor drivers for PAD in relation to the other atherosclerotic manifestations.

The purpose of the present prospective population-based longitudinal study was to evaluate population-attributable risk fractions (PAF) for the four atherosclerotic manifestations and potential differences in risk factor profiles for PAD compared to CoAD, atherothrombotic IS, and CaAD.

## Materials and methods

### Study Population and Data Collection

**Fig. 1:**
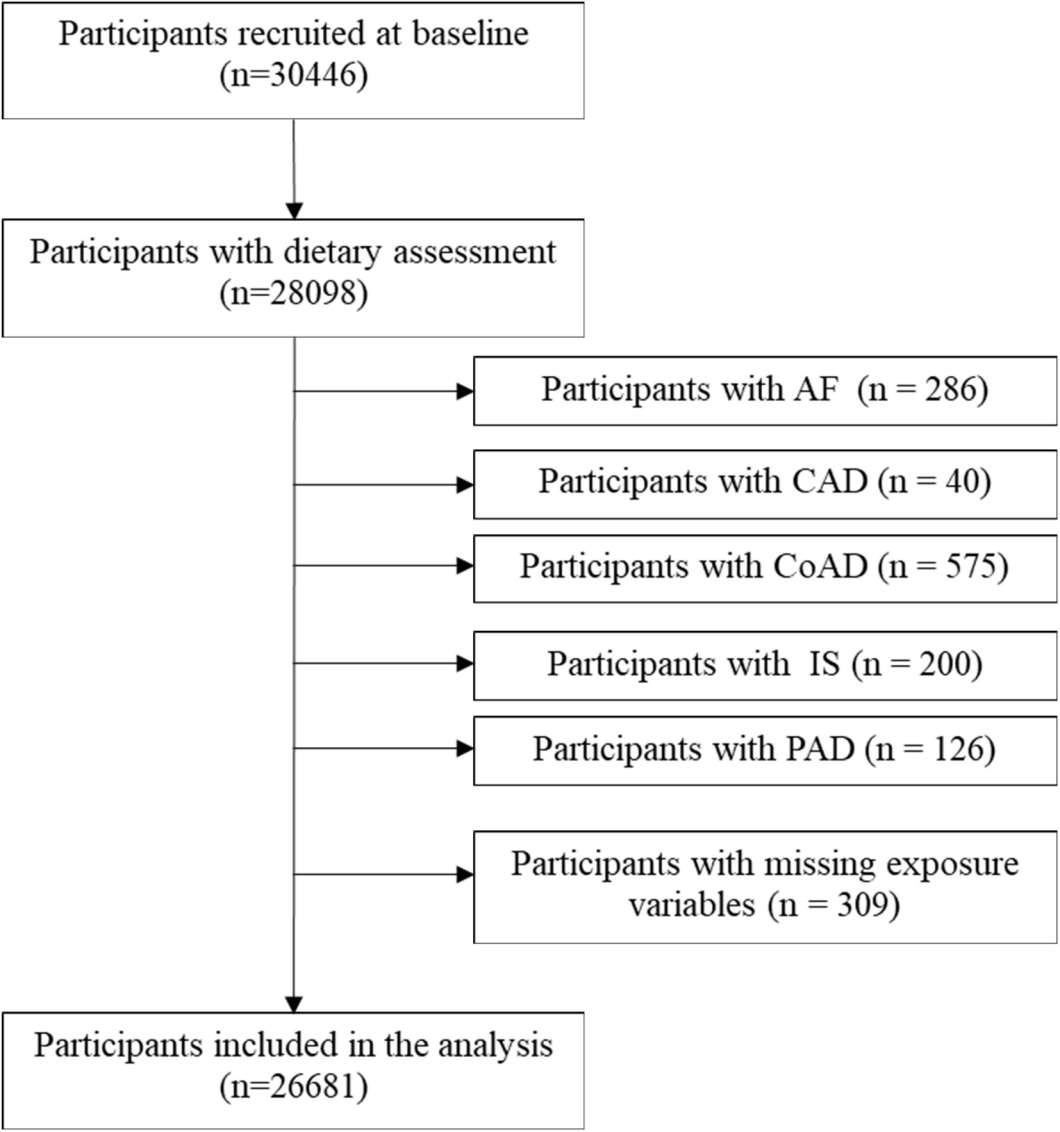
The flowchart of selected participants.

Baseline examinations in the MDCS were carried out between 1991 and 1996 in men and women aged 46–73 years from the background Malmö population^8^. The cohort was followed until 31st December of 2016. Out of the 30,446 participants included, 28,098 participants underwent anthropometric measurements and a dietary assessment. Participants with prevalent atrial flutter or fibrillation (AF), CoAD, IS, CaAD, PAD, and missing exposure variables were excluded, after which 26,681 subjects remained for inclusion in the present study (Fig. 1). The study was conducted ethically in accordance with the 1975 World Medical Association Declaration of Helsinki, and the study protocol was approved by the Ethical Committee at Lund University (Dnr LU 51–90) and the Regional Ethical Review Board in Lund, Sweden (Dnr 2013/566). All subjects gave their written informed consent for participation in the study.

## Diet Assessment Method and Diet Quality Components

The participants filled in a 7-day food diary and a 168-item food frequency questionnaire that included foods regularly consumed the past year. Complementary information was gathered through one-hour interviews. A diet quality index based on the Swedish nutrition recommendations^9^ has been developed and validated within the MDCS cohort^10^. The index includes intake of six dietary components: Saturated fatty acids (SFA) ≤ 14 energy (E)%, polyunsaturated fatty acids (PUFA) 5–10 E%, fish and shellfish ≥ 300 g/week, sucrose ≤ 10 E%, dietary fiber ≥ 2.4 g/megajoule (MJ), fruit and vegetables ≥ 400 g/day. A reached recommendation results in one point per dietary component, with a maximum score of six points. A low score (0–1 points) indicates low quality diet, medium score (2–4 points) medium quality diet and high score (5–6 points) high quality diet^10^.

## Lifestyle and other variables

Data on smoking, alcohol habits, education level, and leisure-time physical activity level was collected via questionnaires. Smoking was defined as never, former, or current smoking. Alcohol intake registered in the 7-day food diary was categorized according to gender specific alcohol limits (quintiles), and zero-consumers were defined as individuals who had not consumed alcohol the past year based on their report in the baseline questionnaire. Leisure-time physical activity level was measured in metabolic equivalent of task hours per week, defined by intensity level and time spent on 17 different activities, adapted from the Minnesota Leisure Time Physical Activity Questionnaire^11^. Body mass index (BMI) ≥ 30 kg/m2 defined obesity. Hypertension was defined as systolic blood pressure ≥ 140 mmHg, diastolic blood pressure ≥ 90 mmHg or current use of antihypertensive medication. DM was defined as having a measured fasting whole blood glucose ≥ 6.1 mmol/L, self-reported history of physician-diagnosed diabetes, use of diabetes medication, or being diagnosed and registered in any of the local or national diabetes registries. Attained education levels were defined as less than 9 years, elementary school (9–10 years), upper secondary school (11–13 years), university without a degree, and university degree.

## Endpoint ascertainment

Incident ASCVD was defined as diagnosis of CoAD, atherothrombotic IS, CaAD or PAD^12^. The Swedish National Patient register^13^ and the Cause of Death Register (http://socialstyrelsen.se/english) were used to identify participants with a first registered diagnosis of ASCVD via civic registration numbers. Diagnoses are coded using a Swedish revision of the International Classification of Disease (ICD), version 8, 9 and 10. Patients registered with atrial fibrillation or flutter (AF) prior to or simultaneously (± 30 days) to ischemic stroke were labeled as having AF related IS and were not labelled as incident atherothrombotic IS. AF related ischemic stroke were followed from baseline up until date of incident AF. AF was ascertained by ICD8-427.9, ICD9-427D and ICD10-I48 codes. Duration of follow up was determined by date of first incident ASCVD for each endpoint, death or end of follow up. The diagnosis of CoAD, atherothrombotic IS, CaAD and PAD in the Swedish National Patient register were separately validated by selecting a random sample of 100 study participants for each diagnosis (Supplementary Material S1).

### Statistical analysis

Baseline characteristics are presented using means ± SDs and percentages for continuous variables and categorical variables, respectively. Because we used survival data, the Direct method was used to estimate PAF^14^. The R package graphPAF was used to estimate population attributable risk fractions (PAF). An age and sex adjusted Cox proportional hazard regression was performed, and the PAF was estimated using the Direct method. The 95% confidence interval (CI) of PAF was estimated using the bootstrap method with 1000 replications. Adjusted hazard ratios (HRs) and 95% CI were estimated by simultaneously entering all risk factors into the Cox proportional hazard regression model. The Multivariable model included sex, age, obesity, hypertension, diabetes mellitus, smoking status, alcohol consumption, leisure-time physical activity, education level, and diet quality score. The proportional hazards assumption was assessed by Schoenfeld´s test and visual inspection of the Schoenfeld residuals, and the assumption was not violated.

To compare the differences in disease and risk factor associations between PAD and three diseases including CoAD, IS, and CaAD, we performed modified Lunn-McNeil^15^ competing risks models. In detail, we first duplicated the dataset so that each individual appeared in two strata and then sorted the failures (PAD-CoAD, PAD-IS, or PAD-CaAD) by strata, and a stratified Cox regression was performed, allowing estimation of separate HRs for two outcomes. Finally, the P value for equal association of a risk factor with two outcomes was estimated by the likelihood ratio test comparing two models with one allowing for the variation of risk factor while another was kept constant. *p* value > 0.05 indicated that the risk factor is equally associated with both outcomes.

SAS, version 9.4 (SAS Institute), and R version 4.2.1 (R Foundation) were used for all analyses. All tests were 2-sided and *p* < 0.05 was considered statistically significant.

## Results

### Baseline characteristics

The median follow-up time for PAD, CoAD, IS and CaAD was 21.6, 21.4, 21.4, and 21.7 years. Baseline data for all participants, divided into those with non-atherosclerotic CV disease, and those with PAD, CoAD, atherothrombotic IS and CaAD, are shown in Table 1.

**Table 1:** Baseline characteristics of the study participants.

	All participants (*n* = 26681)	No incident atherosclerotic CV disease (*n* = 20915)	Incident PAD (*n* = 1089)	Incident CoAD (*n* = 3039)	Incident IS (*n* = 2305)	Incident CaAD (*n* = 476)
Male sex (%)	10,165 (38.1)	7219 (34.5)	547 (50.2)	1690 (55.6)	1072 (46.5)	269 (56.5)
Age (mean ± SD) (years)	57.90 ± 7.59	57.03 ± 7.49	60.78 ± 7.21	61.31 ± 6.99	61.28 ± 7.08	60.23 ± 6.84
Obesity (BMI ≥ 30 kg/m^2^) (%)	3461 (13.0)	2575 (12.3)	160 (14.7)	499 (16.4)	335 (14.5)	65 (13.7)
Hypertension (%)	16,137 (60.5)	11,870 (56.8)	838 (77.0)	2271 (74.7)	1722 (74.7)	360 (75.6)
Diabetes Mellitus (%)	1063 (4.0)	593 (2.8)	128 (11.8)	282 (9.3)	184 (8.0)	39 (8.2)
**Alcohol consumption (%)**						
Zero-consumers	1661 (6.2)	1221 (5.8)	66 (6.1)	235 (7.7)	195 (8.5)	32 (6.7)
Quintile 1	4924 (18.5)	3714 (17.8)	221 (20.3)	664 (21.9)	473 (20.5)	74 (15.6)
Quintile 2	4976 (18.7)	3819 (18.3)	190 (17.5)	629 (20.7)	451 (19.6)	88 (18.5)
Quintile 3	5036 (18.9)	3991 (19.1)	193 (17.7)	546 (18.0)	418 (18.1)	102 (21.4)
Quintile 4	5031 (18.9)	4065 (19.4)	186 (17.1)	496 (16.3)	402 (17.4)	89 (18.7)
Quintile 5	5053 (18.9)	4105 (19.6)	233 (21.4)	469 (15.4)	366 (15.9)	91 (19.1)
**Smoking (%)**						
Never	10,287 (38.6)	8425 (40.3)	198 (18.2)	982 (32.3)	849 (36.8)	114 (24.0)
Former	8849 (33.2)	6995 (33.4)	292 (26.8)	1038 (34.2)	706 (30.6)	173 (36.3)
Current	7545 (28.3)	5495 (26.3)	599 (55.0)	1019 (33.5)	750 (32.5)	189 (39.7)
**Leisure-time physical activity (%)**						
< 7.5 MET-h/week	2550 (9.6)	1894 (9.1)	142 (13.0)	350 (11.5)	257 (11.2)	49 (10.3)
7.5–15.0 MET-h/week	3984 (14.9)	3055 (14.6)	174 (16.0)	489 (16.1)	389 (16.9)	76 (16.0)
15.1–25.0 MET-h/week	6143 (23.0)	4926 (23.6)	239 (22.0)	646 (21.3)	470 (20.4)	101 (21.2)
25.1–50.0 MET-h/week	9716 (36.4)	7735 (37.0)	368 (33.8)	1023 (33.7)	804 (34.9)	158 (33.2)
> 50.0 MET-h/week	4288 (16.1)	3305 (15.8)	166 (15.2)	531 (17.5)	385 (16.7)	92 (19.3)
**Educational level (%)**						
Less than 9 years	11,027 (41.3)	8052 (38.5)	596 (54.7)	1571 (51.7)	1177 (51.1)	231 (48.5)
Elementary school (9–10 y)	7049 (26.4)	5688 (27.2)	239 (22.0)	727 (23.9)	536 (23.3)	126 (26.5)
Elementary + upper secondary school (9–13 y)	2392 (9.0)	1924 (9.2)	84 (7.7)	251 (8.3)	181 (7.9)	36 (7.6)
University studies, no degree	2345 (8.8)	1932 (9.2)	79 (7.3)	218 (7.2)	176 (7.6)	36 (7.6)
University studies, with degree	3868 (14.5)	3319 (15.9)	91 (8.4)	272 (9.0)	235 (10.2)	47 (9.9)
**Diet quality (%)**						
Low	4115 (15.4)	3170 (15.2)	183 (16.8)	469 (15.4)	394 (17.1)	83 (17.4)
Medium	19,013 (71.3)	14,946 (71.5)	788 (72.4)	2141 (70.5)	1619 (70.2)	320 (67.2)
High	3553 (13.3)	2799 (13.4)	118 (10.8)	429 (14.1)	292 (12.7)	73 (15.3)

## Age- and sex-adjusted PAF

As presented in Table 2, current smoking and hypertension were two main factors that contributed to the risk of four manifestations. The proportions of PAD, CoAD, atherothrombotic IS, and CaAD risk attributable to hypertension were 35.1% (95% CI 27.5–42.3), 25.5% (95% CI 19.8–32.8), 27.2% (95% CI 23.8–31.8), and 32.2% (95% CI 25.6–44.1), respectively. Current smoking accounted for 45.6% (95% CI 41.1–47.2), 16.1% (95% CI 14.3–19.0), 14.0% (95% CI 11.5–17.3), and 23.3% (95% CI 15.4–30.0) risk for PAD, CoAD, atherothrombotic IS, and CaAD, respectively. The PAF associated with other risk factors for PAD ranged from 2.1 to 19.1%.

**Table 2:** Age and sex adjusted PAF (%) (95% CI)^a^.

	Incident PAD (*n* = 1089)	Incident CoAD (*n* = 3039)	Incident IS (*n* = 2305)	Incident CaAD (*n* = 476)
Obesity (BMI ≥ 30 kg/m^2^) (%)	2.1 (-0.05, 4.5)	4.4 (2.8, 6.1)	1.9 (0.2, 3.7)	—
Hypertension (%)	35.1 (28.0, 42.0)	25.5 (20.4, 31.0)	27.2 (21.9, 32.7)	32.2 (20.1, 43.6)
Diabetes Mellitus (%)	10.2 (7.6, 12.7)	7.1 (5.7, 8.5)	5.3 (3.9, 6.8)	5.4 (2.0, 8.6)
Current smoking (%)	45.6 (41.9, 49.5)	16.1 (13.6, 18.6)	14.0 (11.4, 16.7)	23.3 (17.2, 29.1)
Highest quintile of alcohol consumption (%)	5.8 (2.8, 8.5)	—	—	—
Low diet quality (diet score 0–1) (%)	3.0 (0.5, 5.6)	1.2 (-0.05, 2.8)	3.2 (1.4, 5.1)	3.9 (-0.5, 8.2)
Sedentary lifestyle (< 7.5 MET-h/week) (%)	5.2 (2.7, 7.6)	3.4 (2.1, 4.7)	3.0 (1.5, 4.5)	—
Lowest level of education (less than 9 years) (%)	19.1 (13.6, 24.4)	12.2 (8.7, 15.6)	11.1 (7.1, 14.8)	—

^**a**^ Confidence interval is computed using bootstrap method with 1000 replications. PAFs for certain exposures and outcomes were not calculated because there were no associations for them according to predefined significance threshold.

### Comparison of risk factors for PAD in relation to CoAD, IS or CaAD

**Fig. 2:**
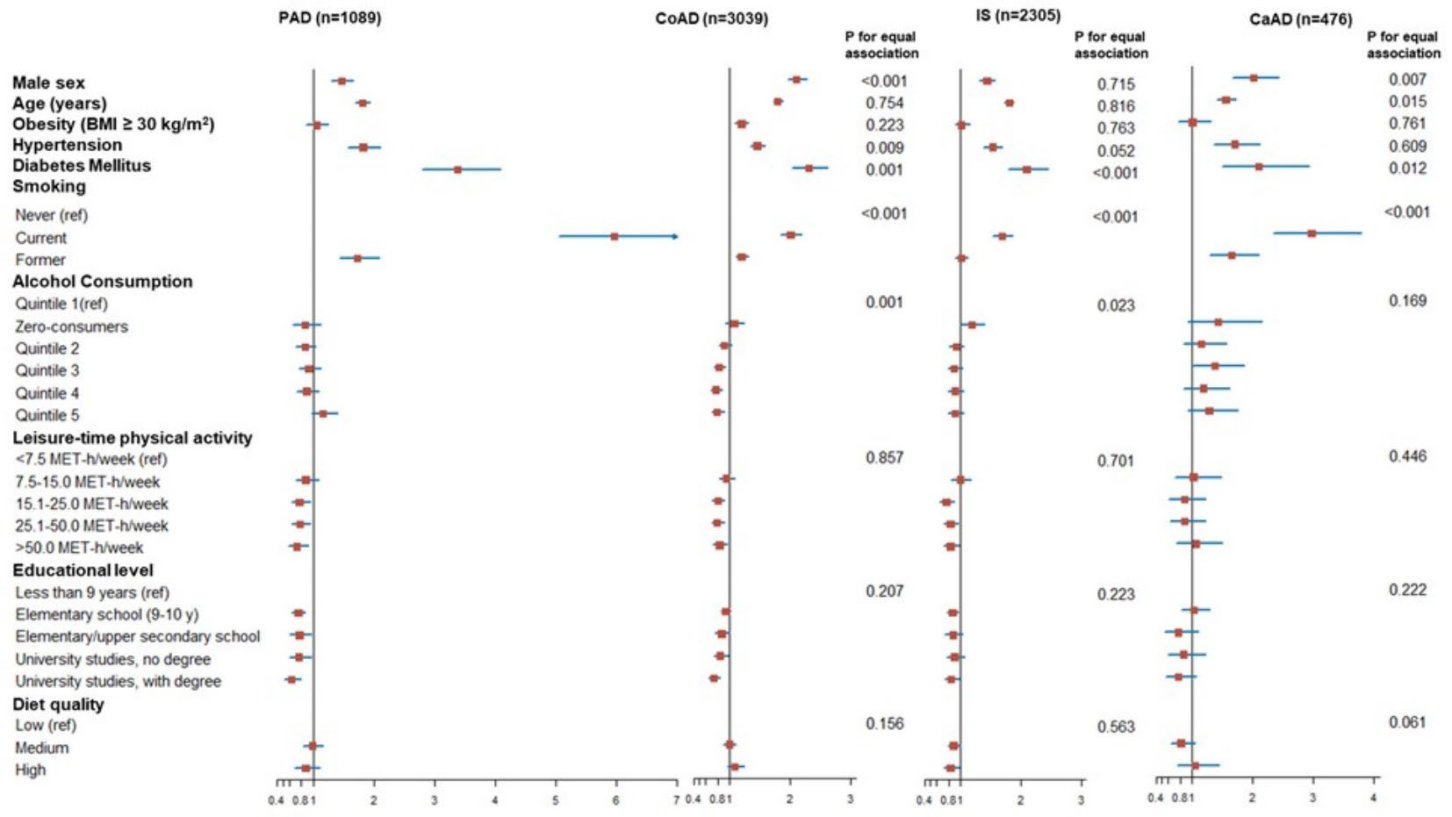
Illustrative forest plot comparing risk factors for PAD, CoAD, IS or CaAD in the Malmö Diet and Cancer Study. Multivariable Cox proportional hazards model included sex, age, obesity, hypertension, diabetes mellitus, smoking, alcohol consumption, physical activity, education, and diet quality score. X-axis represents hazard ratios with 95% confidence intervals. Per one standard deviation increase for age.

Table 3, supplemented with Fig. 2, presents associations of different risk factors with the four atherosclerotic manifestations with associations with PAD as reference. Male sex, age, and hypertension were risk factors for all four manifestations. Compared to PAD risk, the association with male sex was notably stronger for CoAD (*p* < 0.001) and CaAD risk (*p* = 0.007). Hypertension was more associated with PAD than CoAD risk (*p* = 0.009). Smoking and diabetes mellitus were positively associated with all four atherosclerotic manifestations, but the associations were significantly stronger for PAD risk compared to for CoAD risk (*p* < 0.001 and *p* = 0.001, respectively), atherothrombotic IS risk (*p* < 0.001 and *p* < 0.001, respectively) and CaAD risk (*p* < 0.001 and *p* = 0.012, respectively). The inverse associations of higher leisure-time physical activity and educational level with the four atherosclerotic manifestations were similar (*p* > 0.05). Alcohol consumption was unrelated to PAD risk, but inversely associated with CoAD risk (*p* = 0.001 for equal association). Diet Quality was unrelated to PAD risk, but inversely associated with IS risk (*p* = 0.563 for equal association). Stratified analyses of men and women are presented in supplementary tables S2 and S3, respectively. Smoking was more strongly associated with PAD than with the other three atherosclerotic manifestations in both sexes. Diabetes mellitus was more strongly associated with PAD than with the other three atherosclerotic manifestations in men only, whereas there was no difference in strength of associations between diabetes mellitus and different atherosclerotic manifestations in women.

**Table 3:** Comparison of risk factors for PAD, CoAD, IS or CaAD in the Malmö Diet and Cancer Study (*n* = 26681)^a^.

	Incident PAD (*n* = 1089)	Incident CoAD (*n* = 3039)	*P* value for equal association	Incident IS (*n* = 2305)	*P* value for equal association	Incident CaAD (*n* = 476)	*P* value for equal association
Male sex	1.47 (1.30–1.66)	2.10 (1.95–2.27)	< 0.001	1.43 (1.31–1.56)	0.72	2.01 (1.66–2.43)	0.007
**Age (years)** ^**b**^	1.81 (1.69–1.94)	1.79 (1.72–1.87)	0.75	1.80 (1.71–1.88)	0.82	1.55 (1.40–1.72)	0,015
Obesity (BMI ≥ 30 kg/m2)	1.05 (0.88–1.25)	1.19 (1.07–1.31)	0.22	1.01 (0.90–1.14)	0.76	1.00 (0.76–1.30)	0.76
Hypertension	1.82 (1.57–2.11)	1.45 (1.33–1.58)	0.009	1.53 (1.38–1.68)	0.052	1.70 (1.36–2.11)	0.61
Diabetes Mellitus	3.38 (2.80–4.08)	2.30 (2.03–2.61)	0.001	2.09 (1.79–2.44)	< 0.001	2.10 (1.50–2.93)	0.012
**Smoking**							
Never **(ref)**	1 (Ref)	1 (Ref)	< 0.001	1 (Ref)	< 0.001	1 (Ref)	< 0.001
Current	5.97 (5.05–7.05)	2.00 (1.83–2.19)		1.68 (1.52–1.86)		2.97 (2.33–3.78)	
Former	1.73 (1.44–2.08)	1.19 (1.09–1.31)		1.01 (0.91–1.12)		1.65 (1.29–2.10)	
**Alcohol Consumption**							
Quintile 1(ref)	1 (Ref)	1 (Ref)	0.001	1 (Ref)	0.023	1 (Ref)	0.17
Zero-consumers	0.86 (0.65–1.13)	1.07 (0.92–1.24)		1.18 (0.99–1.39)		1.42 (0.93–2.15)	
Quintile 2	0.86 (0.71–1.04)	0.91 (0.82–1.02)		0.92 (0.81–1.05)		1.15 (0.85–1.57)	
Quintile 3	0.92 (0.76–1.12)	0.82 (0.73–0.92)		0.89 (0.78–1.02)		1.37 (1.01–1.85)	
Quintile 4	0.89 (0.73–1.09)	0.77 (0.69–0.87)		0.91 (0.79–1.04)		1.18 (0.86–1.61)	
Quintile 5	1.16 (0.96–1.40)	0.79 (0.70–0.90)		0.91 (0.79–1.04)		1.28 (0.93–1.75)	
**Leisure-time physical activity**							
< 7.5 MET-h/week **(ref)**	1 (Ref)	1 (Ref)	0.86	1 (Ref)	0.70	1 (Ref)	0.45
7.5–15.0 MET-h/week	0.87 (0.70–1.09)	0.94 (0.82–1.08)		0.99 (0.84–1.16)		1.02 (0.71–1.47)	
15.1–25.0 MET-h/week	0.77 (0.63–0.95)	0.80 (0.70–0.91)		0.76 (0.65–0.89)		0.87 (0.62–1.22)	
25.1–50.0 MET-h/week	0.78 (0.64–0.95)	0.79 (0.70–0.90)		0.83 (0.72–0.96)		0.87 (0.63–1.21)	
> 50.0 MET-h/week	0.73 (0.58–0.92)	0.83 (0.72–0.95)		0.83 (0.71–0.97)		1.06 (0.74–1.50)	
**Educational level**							
Less than 9 years **(ref)**	1 (Ref)	1 (Ref)	0.21	1 (Ref)	0.22	1 (Ref)	0.22
Elementary school (9–10 y)	0.75 (0.64–0.87)	0.93 (0.85–1.01)		0.86 (0.77–0.95)		1.03 (0.82–1.28)	
Elementary + upper secondary school (9–13 y)	0.77 (0.61–0.97)	0.86 (0.75–0.99)		0.87 (0.74–1.02)		0.77 (0.54–1.10)	
University studies, no degree	0.76 (0.60–0.96)	0.84 (0.73–0.97)		0.90 (0.77–1.06)		0.85 (0.59–1.21)	
University studies, with degree	0.64 (0.51–0.80)	0.73 (0.64–0.83)		0.84 (0.73–0.97)		0.77 (0.56–1.06)	
**Diet Quality**							
Low **(ref)**	1 (Ref)	1 (Ref)	0.16	1 (Ref)	0.56	1 (Ref)	0.061
Medium	0.98 (0.83–1.15)	0.99 (0.89–1.09)		0.88 (0.79–0.99)		0.81 (0.64–1.04)	
High	0.87 (0.69–1.10)	1.08 (0.95–1.24)		0.83 (0.71–0.97)		1.04 (0.75–1.44)	

^a^ Multivariable Cox proportional hazards model included sex, age, obesity, hypertension, diabetes mellitus, smoking, alcohol consumption, physical activity, education, and diet quality score.

^b^ Per one standard deviation increase.

## Discussion

According to age-and gender adjusted PAF calculations, the present prospective longitudinal cohort study in middle-aged individuals free from atherosclerotic cardiovascular disease and atrial fibrillation at baseline showed that current smoking was the lead risk factor of PAD followed by hypertension. The incidences of PAD and hypertension were estimated to decrease by 45.6% and 35.1%, respectively, in the MDCS population, if elimination of the risk factors current smoking and hypertension were possible. In reverse order, hypertension and current smoking were the most important risk factors for CoAD, atherothrombotic IS, and CaAD. Moreover, the PAF calculation showed that diabetes mellitus (DM) and alcohol consumption were more important risk factors for PAD than for the other atherosclerotic manifestations. Apart from current smoking, the modifiable risk factors obesity, physical activity, and diet contributed little to the overall risk of respective atherosclerotic manifestation, and there was no difference in risk across the four groups.

Age and male sex were associated with a higher risk for all four atherosclerotic manifestations in the present study. The reason for higher risk in male sex may be due to that men have higher life-time risk of ASCVD^16^. Male sex was a greater risk factor for CoAD- and CaAD compared to PAD in the competing risk analysis, which may be related to sex differences regarding social support^17^, clinical presentation, and biological differences such as in CaAD plaque composition^18^. Differences in expression of chest pain, higher proportion of type 1 acute myocardial infarction in men^19^, and higher proportion of type 2 acute myocardial infarction in women^20^ may have resulted in underappreciation of CoAD in women in the present study.

First, it should be noticed that only a very limited number of epidemiological studies have included PAD as an endpoint in addition to cerebral and coronary artery disease. However, an increased risk for PAD related to smoking in comparison to other atherosclerotic manifestations was also found in the Atherosclerosis Risk in Communities (ARIC) Study cohort^21^. A dose-response relationship was identified between pack-years of smoking and PAD, coronary heart disease, and stroke, and the higher risk for PAD appeared after 10–25 pack-years. An elevated risk for PAD persisted up to 30 years following smoking cessation. There was also a longer residual risk for PAD compared to coronary heart disease and stroke up to 5 years after smoking cessation.

The risk for individuals with DM, of which the overwhelming majority had type 2 DM^22^, for development of PAD in the present study was increased by 238%, a figure significantly higher than for the other atherosclerotic manifestations. Patients with DM appear to have a higher likelihood to experience PAD before cardiovascular events arise from other arterial beds. In a retrospective cohort study of middle-aged patients with type 1 DM without prior CV events, PAD was the most frequent first CV event occurring in 39.5% of all individuals, whereas ischemic heart disease and cerebral vascular disease occurred first in 29.5% and 16.6%, respectively^23^. In a prospective hospital-based cross-sectional study on type 2 DM patients, the rate of microvascular complications was very high; retinopathy, neuropathy, and nephropathy were documented in 51.4%, 77.1%, and 30.5%, respectively^24^. Hence, consequences of DM seem to affect the macrovascular and microvascular circulation in the lower extremity arterial system first. A recent epidemiological review on poly-vascular disease concluded that smoking and DM are both associated with a relatively higher risk for PAD in relation to CoAD and stroke^25^. The risk for PAD is further increased if individuals with DM have been exposed to smoking^26^. In contrast to general population screening, individual patients with high-risk features such as DM and smoking, might benefit from identification of PAD and more aggressive medical risk factor control management^27^. A systematic review and meta-analysis of the diagnostic accuracy of bedside tests to establish the presence of PAD in people with DM documented the efficacy of toe-brachial pressure index and tibial artery waveform assessment^28^, whereas ankle-brachial pressure index had a too poor sensitivity^28,29^. Development and evaluation of better screening tools for PAD in individuals with DM is therefore warranted.

The gender-stratified analyses showed that diabetes mellitus appeared to be a stronger risk factor for development of PAD compared to the other three atherosclerotic manifestations in men, whereas diabetes mellitus conferred the same increases in risk for all four atherosclerotic manifestations in women. A plausible explanation for this discrepancy between sexes might be underdiagnosis of PAD in women, as they appear to seek medical attention in more advanced stages of PAD than men^30^. The misconception that PAD is predominantly found in men^31^, and that women have higher rates of asymptomatic and atypical PAD^32^, might contribute to lack of recognition of PAD in women. However, a recent meta-analysis of six prospective cohort studies did not find an excess risk of PAD in any of the sexes^33^. The low number of cohort studies in this meta-analysis and the use of the unsensitive ankle-brachial pressure index to screen for PAD in the included studies^33^ call for the need of increased reporting of sex-specific results in PAD research, and for more accurate tools to screen for PAD at the toe level in individuals with diabetes mellitus.

Compared to PAD risk, medium and high level of alcohol consumption was associated with reduced CoAD risk in the present study. The highest category of alcohol consumption was defined as slightly more than one glass of wine (12 cl) per day in women and slightly more than two glasses of wine per day in men. Several cohort studies have shown an inverse association between alcohol consumption and PAD^34–38^. Low to moderate alcohol consumption (< 30 g per day) was found to confer the strongest risk reduction for CoAD in meta-analysis^39^. In another systematic review and meta-analysis, however, low and moderate alcohol consumption was inversely associated only with ischemic stroke, whereas heavy drinking was associated with increased risk of all stroke types with a strong association for hemorrhagic strokes^40^. Putative protective mechanisms against development of atherosclerosis in subjects with low to moderate alcohol consumption are inhibition of atheroma formation, decreasing of blood coagulation through increased levels of high-density lipoprotein cholesterol, decreased levels of low-density lipoproteins cholesterol, prevention of clot formation, reduction in platelet aggregation^41^, and lower levels of fibrinogen^42^. A large epidemiological study in a Chinese population using conventional and genetic evidence, showed that conventional epidemiology using self-reported alcohol intake in men had U-shaped associations with incidence of stroke and myocardial infarction, while genetic epidemiological study using genetic variants that greatly reduces breakdown of acetaldehyde, resulting in accumulation in blood, severe discomfort and, consequently strongly reduces alcohol intake, have shown that alcohol consumption uniformly increases blood pressure and stroke risk in men without any U-shaped associations with risk^43^.

Differences in risk factor profiles between the arterial beds cannot easily be translated into different pathogenic mechanistic pathways. The pathological disparities between, for instance PAD and CoAD, may be speculated upon: A cross-sectional study found that active smoking and higher levels of CRP have a greater role in PAD^44^, suggesting a more pronounced inflammatory activity in this arterial bed. The greater role of hypertension in PAD compared to CoAD in the present study might be related to differences in responses of platelets to fluid shear stress^45^ and perhaps development of luminal thrombi more associated with insignificant atherosclerosis suggesting the possibility of atherothromboembolic disease in PAD^46^, whereas thrombosis in CoAD occurs secondary to disruption and erosion of the fibrous cap of atheromatous plaques^47^. A genome-wide association study suggests that there are genetic loci common for disease in the coronary, cerebral and peripheral arterial beds, and loci variants specific for PAD involved in pathways promoting thrombosis, providing genetic support for Factor Xa inhibition as a therapeutic strategy for PAD^48^.

There are some limitations of the study. Self-reported dietary intake data are likely to be associated with some degree of misclassification and may have changed during follow up. Data on the average number of cigarettes per day or pack-years was not available. Repeated measurements of life-style modifiable risk factors for atherosclerotic disease would have been valuable, since for instance smoking prevalence has decreased significantly in the last decades in Sweden^49^. Blood testing for Hemoglobin A1c, a marker of glycemic control and an independent risk factor for macro- and microvascular complications in DM^50,51^, was only available at baseline in a subset of MDCS patients^52^. Other limitations of the study are the absence of ethnicity and biochemical data resulting in unmeasured confounding, and as always residual confounding coherent with the study design. Since the participation rate of the study was 40% of middle-aged Malmö citizens, and participants reported better health than non-participants^53^, external validity of study results to the general population should be regarded as low to intermediate, whereas the generalizability to similar cohort populations may be high. The large sample size of middle-aged individuals, the long duration of follow up, and the validation of atherosclerotic disease endpoints are major strengths of this study. The calculation of PAF adjusted for age and gender, and the competing risk approach for calculating differences in HR between two groups of atherosclerotic disease represent other study strengths.

It should be noted that mainly incident symptomatic cases of PAD, CoAD and athero-thrombotic IS were identified in this study, whereas there was a rather large proportion of asymptomatic cases among patients with incident CaAD. Incident PAD manifested itself as severe ischemia in 82% of cases, intermittent claudication in 15%, and asymptomatic in only 1%. In a population-based study on elderly subjects of PAD prevalence in Sweden, on the other hand, asymptomatic PAD (diagnosed as low ankle-brachial index only), intermittent claudication and severe limb ischemia were prevalent in 11%, 7% and 1.2%, respectively^54^. Hence, it is likely that substantially more participants in the MDCS would have been diagnosed with asymptomatic PAD and probably also intermittent claudication if systematically examined with ABI. This raises the question of targeted PAD screening with ABI measurements in subjects exposed to smoking, hypertension, and diabetes mellitus.

Atherosclerosis is a systemic disease involving multiple arterial beds. The present prospective study has shown that risk factor profiles vary for the different arterial territories. Smoking and diabetes mellitus had a larger impact on incident PAD than the other three atherosclerotic manifestations, which may have implications for screening for PAD^55^ in these individuals at increased risk. A prospective study supplemented with laboratory data originally designed to study atherosclerotic manifestations in the different arterial beds is warranted.

In conclusion, smoking and DM have a larger impact on incident PAD than on incident coronary, cerebral or precerebral artery manifestations, and hypertension were more associated with incident PAD than with incident CoAD. Since the lower extremity arteries are the easiest to access and examine, they may be considered as the first arterial bed to examine in patients at increased risk for atherosclerotic manifestations.

## Electronic supplementary material

Below is the link to the electronic supplementary material.

Supplementary Material 1

## Data Availability

Datasets analyzed during the current study are not publicly available due to the nature of sensitive personal data and study materials. However, procedures for sharing data, analytic methods, and study materials for reproducing the results following Swedish legislation can be arranged by contacting the corresponding author or study organization (https://www.malmo-kohorter.lu.se/malmo-kost-cancer-mkc).
