# Supplementary material for: Differences in risk factor profiles for peripheral artery disease compared to coronary, cerebral and carotid artery
Source: Sci Rep. 2025 Jan 31;15:3864. doi: 10.1038/s41598-025-88516-0 (PMC11785722; doi:10.1038/s41598-025-88516-0)
Supplement: Supplementary file 1 — Supplementary Material 1 [file 41598_2025_88516_MOESM1_ESM.docx]

**Supplementary Material S1. Validation of diagnosis of atherosclerotic cardiovascular disease**

*Validation of diagnosis of coronary artery disease*

One hundred patients with diagnosis of coronary artery disease were randomly selected for the validation procedure using patient record data. Among 100 patients, 96 had acute myocardial infarction, of which 29 had ST-Elevation Myocardial Infarction (STEMI) and 67 had non-STEMI (NSTEMI). One patient died due to acute circulatory insufficiency due to unclear cause, but did not undergo autopsy. Three patients did not have acute myocardial infarction; one had transitory cerebral ischemic attack, one had acute pulmonary embolism, and one had acute biliary duct stone disease. Hence, acute myocardial infarction was confirmed in 96% of cases.

**Diagnostic codes for coronary artery disease**

|  | **ICD-8 ( -1986)** | **ICD-9 (1987-96)** | **ICD-10 (1997- )** |
| --- | --- | --- | --- |
| **Coronary artery disease** | **410** | **410A – 410X** | **I21** |

*Validation of diagnosis of ischemic stroke*

One hundred patients with diagnosis of ischemic were randomly selected for the validation procedure using patient record data. Among 100 patients, 89 had stroke and 87 had IS. Two patients had intra-cerebral hemorrhage. It was unclear if one patient with fatal outcome had stroke or not, and autopsy was not undertaken. Of the ten patients that did not have stroke, four had transitory ischemic attack due to intra-cerebral thrombosis. Six patients did not have a cerebral ischemic event due to epilepsy (n=1), primary progressive aphasia (n=1), syncope (n=1), disorientation (n=1), headache (n=1) and acute lower limb ischemia (n=1). Among 87 with ischemic stroke, the distribution of causes were the following: Intra-cerebral thrombosis (n=43; 49.4%), embolization secondary to atrial fibrillation (n=31; 35.6%), embolization due to carotid artery stenosis (n=7; 8.0%), carotid artery dissection (n=2), embolization secondary to endocarditis (n=1), unclear if symptomatic carotid artery stenosis or intra-cerebral thrombosis (n=2) and unclear if cardiac arrhythmias or intra-cerebral thrombosis (n=1). Among the 98 evaluable patients, 56 (57%) had an atherosclerotic cause of disease. The diagnosis of ischemic stroke was confirmed in 89% (87/98) of cases.

**Diagnostis codes for ischemic stroke**

|  | **ICD-8 ( -1986)** | **ICD-9 (1987-96)** | **ICD-10 (1997- )** |
| --- | --- | --- | --- |
| **Ischemic stroke** | **433,434** | **434, 436** | **I63, I64** |

*Validation of diagnosis of carotid artery disease diagnosis*

One hundred patients with diagnosis of carotid artery disease were randomly selected for the validation procedure using patient record data. Among 100 patients, 57 had symptomatic (≥ 60% stenosis of the internal carotid artery on color doppler ultrasound) and 42 asymptomatic carotid artery disease. The proportion of operated patients with symptomatic and asymptomatic (≥ 70%) carotid artery disease was 85.9% (49/57) and 14.3% (6/42), respectively. One patient had coronary artery disease and was misdiagnosed. The diagnosis of carotid artery disease was therefore confirmed in 99% of the validation sample and symptomatic carotid artery disease in 57%.

**Diagnostic codes for carotid artery disease**

|  | **ICD-8 ( -1986)** | **ICD-9 (1987-96)** | **ICD-10 (1997- )** |
| --- | --- | --- | --- |
| **Occlusion or stenosis of carotid artery** | **432,00**  **432,90** | **433B** | **I65.2** |

*Validation of diagnosis of peripheral artery disease*

One hundred patients with diagnosis of peripheral artery disease were randomly selected for the validation procedure using patient record data. Among 100 patients, 69 had critical limb ischemia, 13 had acute limb ischemia, 15 had intermittent claudication, and one had asymptomatic peripheral artery disease. Of the 13 patients with acute limb ischemia, 12 had acute thrombotic occlusion and one had an embolic occlusion. Two patients had venous insufficiency and were thus misdiagnosed. The diagnosis of peripheral artery disease could therefore be confirmed in 98% of cases and symptomatic peripheral artery disease in 97%.

**Diagnostic codes for peripheral arterial disease**

|  | **ICD-8 ( -1986)** | **ICD-9 (1987-96)** | **ICD-10 (1997- )** |
| --- | --- | --- | --- |
| **Claudicatio** | **443,90**  **443,99** | **443X** | **I73.9 (all subcodes, except I73.9A)** |
| **Critical limb ischaemia** | **445,00**  **445,98**  **445,99** | **440C** | **I70.2 (all subcodes)** |

| **Supplementary Table S2a. Comparison of risk factors for PAD, CoAD, IS or CaAD in the Malmö Diet and Cancer Study among men (n = 10165)^a^** | | | | | | | |
| --- | --- | --- | --- | --- | --- | --- | --- |
|  | **Incident PAD (n=547)** | **Incident CoAD (n=1690)** | ***P* value for equal association** | **Incident IS (n=1072)** | ***P* value for equal association** | **Incident CaAD (n=269)** | ***P* value for equal association** |
| **Age (years)^b^** | 1.73 (1.56-1.92) | 1.65 (1.56-1.75) | 0.42 | 1.70 (1.58-1.83) | 0.78 | 1.55 (1.34-1.78) | 0.21 |
| Obesity (BMI ≥ 30 kg/m2) | 1.15 (0.90-1.46) | 1.16 (1.01-1.33) | 0.93 | 1.09 (0.92-1.31) | 0.76 | 0.93 (0.64-1.36) | 0.36 |
| Hypertension | 1.83 (1.48-2.27) | 1.36 (1.21-1.52) | 0.014 | 1.46 (1.26-1.69) | 0.083 | 1.49 (1.12-1.99) | 0.26 |
| Diabetes Mellitus | 3.72 (2.92-4.74) | 2.03 (1.72-2.40) | <0.001 | 2.03 (1.64-2.52) | <0.001 | 2.25 (1.49-3.39) | 0.033 |
| **Smoking** |  |  |  |  |  |  |  |
| Never **(ref)** | 1 (Ref) | 1 (Ref) | <0.001 | 1 (Ref) | <0.001 | 1 (Ref) | <0.001 |
| Current | 6.92 (5.19-9.23) | 1.95 (1.71-2.22) |  | 1.64 (1.39-1.92) |  | 3.19 (2.21-4.59) |  |
| Former | 2.26 (1.68-3.03) | 1.21 (1.07-1.37) |  | 1.07 (0.92-1.25) |  | 1.94 (1.37-2.76) |  |
| **Alcohol Consumption** |  |  |  |  |  |  |  |
| Quintile 1(ref) | 1 (Ref) | 1 (Ref) | 0.12 | 1 (Ref) | 0.17 | 1 (Ref) | 0.34 |
| Zero-consumers | 0.71 (0.42-1.18) | 0.98 (0.77-1.25) |  | 1.43 (1.07-1.90) |  | 1.37 (0.70-2.67) |  |
| Quintile 2 | 0.81 (0.62-1.06) | 0.88 (0.76-1.02) |  | 0.98 (0.80-1.18) |  | 1.19 (0.78-1.80) |  |
| Quintile 3 | 0.84 (0.64-1.10) | 0.74 (0.64-0.87) |  | 0.94 (0.77-1.15) |  | 1.42 (0.95-2.12) |  |
| Quintile 4 | 0.84 (0.64-1.10) | 0.72 (0.62-0.85) |  | 1.06 (0.87-1.28) |  | 1.21 (0.80-1.84) |  |
| Quintile 5 | 1.02 (0.78-1.32) | 0.76 (0.65-0.89) |  | 1.03 (0.84-1.27) |  | 1.33 (0.88-2.01) |  |
| **Leisure-time physical activity** |  |  |  |  |  |  |  |
| <7.5 MET-h/week **(ref)** | 1 (Ref) | 1 (Ref) | 0.61 | 1 (Ref) | 0.97 | 1 (Ref) | 0.85 |
| 7.5-15.0 MET-h/week | 0.95 (0.70-1.30) | 1.07 (0.88-1.30) |  | 0.87 (0.69-1.09) |  | 0.91 (0.57-1.46) |  |
| 15.1-25.0 MET-h/week | 0.76 (0.56-1.03) | 0.93 (0.78-1.12) |  | 0.70 (0.56-0.87) |  | 0.74 (0.47-1.17) |  |
| 25.1-50.0 MET-h/week | 0.77 (0.58-1.03) | 0.89 (0.75-1.06) |  | 0.70 (0.57-0.86) |  | 0.82 (0.54-1.25) |  |
| >50.0 MET-h/week | 0.73 (0.53-1.01) | 0.98 (0.81-1.18) |  | 0.75 (0.60-0.94) |  | 0.91 (0.57-1.43) |  |
| **Educational level** |  |  |  |  |  |  |  |
| Less than 9 years **(ref)** | 1 (Ref) | 1 (Ref) | 0.093 | 1 (Ref) | 0.26 | 1 (Ref) | 0.25 |
| Elementary school (9-10 y) | 0.75 (0.59-0.94) | 1.02 (0.90-1.16) |  | 0.85 (0.72-1.01) |  | 1.09 (0.79-1.48) |  |
| Elementary + upper secondary school (9-13 y) | 0.74 (0.55-0.99) | 0.92 (0.78-1.08) |  | 0.93 (0.76-1.13) |  | 0.91 (0.61-1.37) |  |
| University studies, no degree | 0.72 (0.52-1.00) | 0.87 (0.72-1.04) |  | 0.87 (0.69-1.09) |  | 1.03 (0.67-1.59) |  |
| University studies, with degree | 0.56 (0.41-0.77) | 0.78 (0.66-0.92) |  | 0.82 (0.67-1.01) |  | 0.84 (0.56-1.27) |  |
| **Diet Quality** |  |  |  |  |  |  |  |
| Low **(ref)** | 1 (Ref) | 1 (Ref) | 0.51 | 1 (Ref) | 0.78 | 1 (Ref) | 0,.64 |
| Medium | 0.83 (0.66-1.04) | 0.96 (0.84-1.10) |  | 0.79 (0.67-0.93) |  | 0.71 (0.52-0.98) |  |
| High | 0.88 (0.63-1.23) | 1.08 (0.89-1.30) |  | 0.76 (0.60-0.96) |  | 0.89 (0.57-1.38) |  |
| ^a^ Multivariable Cox proportional hazards model included sex, age, obesity, hypertension, diabetes mellitus, smoking, alcohol consumption, physical activity, education, and diet quality score.  ^b^ Per one standard deviation increase. | | | | | | | |

| **Supplementary Table S2b. Comparison of risk factors for PAD, CoAD, IS or CaAD in the Malmö Diet and Cancer Study among women (n = 16516)^a^** | | | | | | | |
| --- | --- | --- | --- | --- | --- | --- | --- |
|  | **Incident PAD (n=542)** | **Incident CoAD (n=1349)** | ***P* value for equal association** | **Incident IS (n=1233)** | ***P* value for equal association** | **Incident CaAD (n=207)** | ***P* value for equal association** |
| Age (years)^b^ | 1.90 (1.73-2.09) | 1.93 (1.82-2.06) | 0.76 | 1.87 (1.76-2.00) | 0.81 | 1.52 (1.30-1.77) | 0.016 |
| Obesity (BMI ≥ 30 kg/m2) | 0.97 (0.76-1.24) | 1.18 (1.02-1.36) | 0.17 | 0.95 (0.81-1.11) | 0.89 | 1.06 (0.72-1.56) | 0.70 |
| Hypertension | 1.79 (1.47-2.20) | 1.55 (1.36-1.77) | 0.24 | 1.57 (1.37-1.79) | 0.27 | 1.97 (1.41-2.74) | 0.64 |
| Diabetes Mellitus | 2.95 (2.17-4.01) | 2.72 (2.25-3.29) | 0.66 | 2.16 (1.73-2.70) | 0.11 | 1.82 (1.02-3.23) | 0.13 |
| **Smoking** |  |  |  |  |  |  |  |
| Never **(ref)** | 1 (Ref) | 1 (Ref) | <0.001 | 1 (Ref) | <0.001 | 1 (Ref) | <0.001 |
| Current | 5.62 (4.56-6.92) | 2.09 (1.84-2.38) |  | 1.76 (1.54-2.01) |  | 2.92 (2.10-4.05) |  |
| Former | 1.33 (1.02-1.74) | 1.21 (1.06-1.39) |  | 0.96 (0.83-1.12) |  | 1.39 (0.96-2.01) |  |
| **Alcohol Consumption** |  |  |  |  |  |  |  |
| Quintile 1(ref) | 1 (Ref) | 1 (Ref) | 0.053 | 1 (Ref) | 0.047 | 1 (Ref) | 0.73 |
| Zero-consumers | 0.98 (0.69-1.38) | 1.12 (0.92-1.37) |  | 1.05 (0.86-1.30) |  | 1.40 (0.81-2.41) |  |
| Quintile 2 | 0.90 (0.68-1.19) | 0.97 (0.82-1.15) |  | 0.88 (0.74-1.04) |  | 1.11 (0.70-1.76) |  |
| Quintile 3 | 1.01 (0.76-1.34) | 0.93 (0.78-1.10) |  | 0.86 (0.72-1.03) |  | 1.30 (0.83-2.05) |  |
| Quintile 4 | 0.94 (0.70-1.26) | 0.86 (0.71-1.03) |  | 0.78 (0.65-0.94) |  | 1.12 (0.70-1.81) |  |
| Quintile 5 | 1.30 (0.99-1.71) | 0.85 (0.70-1.03) |  | 0.79 (0.65-0.97) |  | 1.22 (0.75-1.98) |  |
| **Leisure-time physical activity** |  |  |  |  |  |  |  |
| <7.5 MET-h/week **(ref)** | 1 (Ref) | 1 (Ref) | 0.85 | 1 (Ref) | 0.51 | 1 (Ref) | 0.35 |
| 7.5-15.0 MET-h/week | 0.80 (0.58-1.11) | 0.82 (0.67-0.99) |  | 1.12 (0.90-1.39) |  | 1.21 (0.69-2.12) |  |
| 15.1-25.0 MET-h/week | 0.78 (0.58-1.05) | 0.67 (0.56-0.81) |  | 0.84 (0.67-1.04) |  | 1.07 (0.63-1.82) |  |
| 25.1-50.0 MET-h/week | 0.78 (0.59-1.03) | 0.72 (0.60-0.85) |  | 0.98 (0.80-1.20) |  | 0.96 (0.57-1.61) |  |
| >50.0 MET-h/week | 0.73 (0.52-1.02) | 0.71 (0.58-0.88) |  | 0.92 (0.73-1.16) |  | 1.35 (0.77-2.35) |  |
| **Educational level** |  |  |  |  |  |  |  |
| Less than 9 years **(ref)** | 1 (Ref) | 1 (Ref) | 0.81 | 1 (Ref) | 0.70 | 1 (Ref) | 0.27 |
| Elementary school (9-10 y) | 0.75 (0.61-0.93) | 0.84 (0.74-0.96) |  | 0.87 (0.76-1.00) |  | 0.96 (0.70-1.32) |  |
| Elementary + upper secondary school (9-13 y) | 0.86 (0.59-1.26) | 0.80 (0.61-1.04) |  | 0.79 (0.60-1.04) |  | 0.50 (0.23-1.08) |  |
| University studies, no degree | 0.82 (0.58-1.16) | 0.81 (0.65-1.03) |  | 0.94 (0.75-1.18) |  | 0.62 (0.33-1.17) |  |
| University studies, with degree | 0.75 (0.54-1.03) | 0.67 (0.54-0.84) |  | 0.89 (0.72-1.09) |  | 0.68 (0.40-1.15) |  |
| **Diet Quality** |  |  |  |  |  |  |  |
| Low **(ref)** | 1 (Ref) | 1 (Ref) | 0.077 | 1 (Ref) | 0.26 | 1 (Ref) | 0.060 |
| Medium | 1.16 (0.92-1.47) | 1.01 (0.87-1.17) |  | 0.97 (0.83-1.13) |  | 0.96 (0.66-1.41) |  |
| High | 0.88 (0.63-1.24) | 1.08 (0.89-1.31) |  | 0.90 (0.73-1.11) |  | 1.27 (0.79-2.04) |  |
| ^a^ Multivariable Cox proportional hazards model included sex, age, obesity, hypertension, diabetes mellitus, smoking, alcohol consumption, physical activity, education, and diet quality score.  ^b^ Per one standard deviation increase. | | | | | | | |
